# Evaluation of antitumor activity of a TGF-beta receptor I inhibitor (SD-208) on human colon adenocarcinoma

**DOI:** 10.1186/2008-2231-22-47

**Published:** 2014-06-05

**Authors:** Abolfazl Akbari, Saeid Amanpour, Samad Muhammadnejad, Mohammad Hossein Ghahremani, Seyed Hamidollah Ghaffari, Ahmad Reza Dehpour, Gholam Reza Mobini, Fatemeh Shidfar, Mahdi Abastabar, Ahad Khoshzaban, Ebrahim Faghihloo, Abbas Karimi, Mansour Heidari

**Affiliations:** 1 Department of Molecular Medicine, School of Advanced Medical Technologies, Tehran University of Medical Sciences, Tehran, Iran; 2 Cancer Research Center, Cancer Institute of Iran, Tehran University of Medical Sciences, Tehran, Iran; 3 Department of Pharmacology and Toxicology, Faculty of Pharmacy, Tehran University of Medical Sciences, Tehran, Iran; 4 Hematology, Oncology and Stem Cell Transplantation Research Center, Shariati Hospital, Tehran University of Medical Sciences, Tehran, Iran; 5 Department of Pharmacology, School of Medicine, Tehran University of Medical Sciences, Tehran, Iran; 6 Invasive Fungi Research Center, Department of Medical Mycology and Parasitology, School of Medicine, Mazandaran University of Medical Sciences, Sari, Iran; 7 Stem Cells Preparation Uinte, Farabi Eye Hospital, Tehran University of Medical Sciences, Tehran, Iran; 8 Department of Medical Genetics, School of Medicine, Tehran University of Medical Sciences, Tehran, Iran; 9 Department of Virology, School of Public Health, Tehran University of Medical Sciences, Tehran, Iran; 10 Department of Molecular Medicine, Faculty of Advanced Technologies in Medicine (FATiM), Iran University of Medical Sciences, Tehran, Iran

**Keywords:** SD-208, Colorectal cancer, SW-48, Immunohistochemistry staining

## Abstract

**Background:**

Transforming growth factor-β (TGF-β) pathway is involved in primary tumor progression and in promoting metastasis in a considerable proportion of human cancers such as colorectal cancer (CRC). Therefore, blockage of TGF-β pathway signaling via an inhibitor could be a valuable tool in CRC treatment.

**Methods:**

To evaluate the efficacy of systemic targeting of the TGF-β pathway for therapeutic effects on CRC, we investigated the effects of a TGβRI (TGF-β receptor 1) or TβRI kinase inhibitor, SD-208, on SW-48, colon adenocarcinoma cells. In this work, *in vitro* cell proliferation was studied by methyl thiazolyl tetrazolium (MTT) and bromo-2′-deoxyuridine (BrdU) assays. Also, the histopathological and immunohistochemical evaluations were conducted by hematoxylin and eosin, and Ki-67 and CD34 markers were stained, respectively.

**Results:**

Our results showed no significant reduction in cell proliferation and vessel formation (170 ± 70 and 165 ± 70, P > 0.05) in treated SW-48 cells with SD-208 compared to controls.

**Conclusion:**

Our data suggested that SD-208 could not significantly reduce tumor growth and angiogenesis in human colorectal cancer model at least using SW-48 cells.

## Background

Colorectal cancer (CRC) is the second leading cause of cancer death among adults, making it as an excellent and desirable area for clinicians and researchers to study [1,2]. It has been also found that a large proportion of cancers such as CRC display inactivation of the growth factors specially transforming growth factor-β (TGF-β) pathway but they are characterized by increasing the factor production. In man, three isoforms of TGF-β including TGF-β1, TGF-β2 and TGF-β3 have been well characterized [3,4]. The homology searching has revealed that these proteins share up to 75% amino acid sequences. In spite of the fact that they have demonstrated comparable signaling activities, they are differentially expressed in cell lineages and tissues. It has been also frequently reported that TGF-β is the most effective growth inhibitor for normal epithelial, hematopoietic and immune cells. Studies showed that TGF-β acts as a double-edged sword in the biological processes [3-6]. In addition, it plays important roles in primary tumor progression and in promoting metastasis and has become an attractive target for therapy [5-7]. TGF-β exerts its effects by acting on two types of transmembrane receptors: type I (TGβRI) and type II (TGβRII). These receptors are involved in signal transduction, triggers through interaction of the TGF-β with TGβRII. Once TGF-β binds to TGβRII leads to the recruitment, phosphosphorylation, and activation of TGβRI. Activation of the TGβRI invokes several TGF-β signaling pathway targets and downstream genes [7,8]. Based on the literature, targeting of TGF-β signaling could be a valuable tool to treatment of human cancers such as CRC, glioblastoma and breast cancer [6-10]. Several studies reported that a series of pyridopyrimidine-based TGβRI kinase inhibitors such as SD-208 could be a powerful approach for the treatment of various cancers. SD-208 is a small-molecule kinase inhibitor that bind to the ATP-binding site of the TGβRI kinase and maintains the enzyme in its inactive configuration [11-13]. Despite advances in our understanding of the molecular and genetic basis of CRC, effective treatment of the disease remains a clinical challenge. As pointed out, TGF-β promotes cell proliferation, development, invasion and metastasis in several types of tumors. It is thought that TGβRI inhibitor may have a therapeutic benefit in CRC; therefore, pharmacological blockade of the TGF-β signaling pathway has been proposed as a benefit strategy for CRC therapy. In the current study, SW-48, colon adenocarcinoma cell line was treated with different doses of SD-208, an anti-cancer agent. We evaluated the effects of SD-208 on cell proliferation and differentiation *in vitro* and *in vivo*.

## Methods

### Cell culture

Human colorectal adenocarcinoma cell line with pathologic differentiation grade of the original tumors IV, SW-48, was obtained from National Cell Bank of Iran (NCBI) affiliated to Pasteur Institute (Tehran, Iran). The cell line was grown in RPMI-1640 medium (Gibco; Germany) containing 25 mM D-glucose, 4 mM L-glutamine and 1 mM sodium pyruvate and supplemented with 5% (v/v) heat inactivated fetal bovine serum (FBS) (Gibco; Germany), 2 mM glutamax (Gibco; Germany), 100 units/ml penicillin, 100 μg/ml streptomycin and 250 ng/ml amphoterycin (Gibco; Germany) in culture flask 25 cm^2^ (SPL Life Sciences; South Korea). The cells were kept at 37°C in a humidified 95% atmosphere, 5% CO_2_ atmosphere incubator designated as culture at a steady-state condition. Cell viability was assessed using trypan blue exclusion test and routinely found to all flasks contain more than 95% viable cells.

### Chemical description and biological activity TGβRI kinase inhibitor, SD-208

SD-208 (Sigma Aldrich; Belgium) is a selective and orally active pyridopyrimidine type TGβRI kinase inhibitor with an IC50 of approximately 35 nmol/L against TβRI kinase activity *in vitro*. The drug was dissolved in 100% dimethylsulfoxide (DMSO; Sigma Aldrich; Belgium) and prepared as stock solutions of 5 mM in DMSO and kept at −20°C until use.

### MTT assay

**Figure 1:**
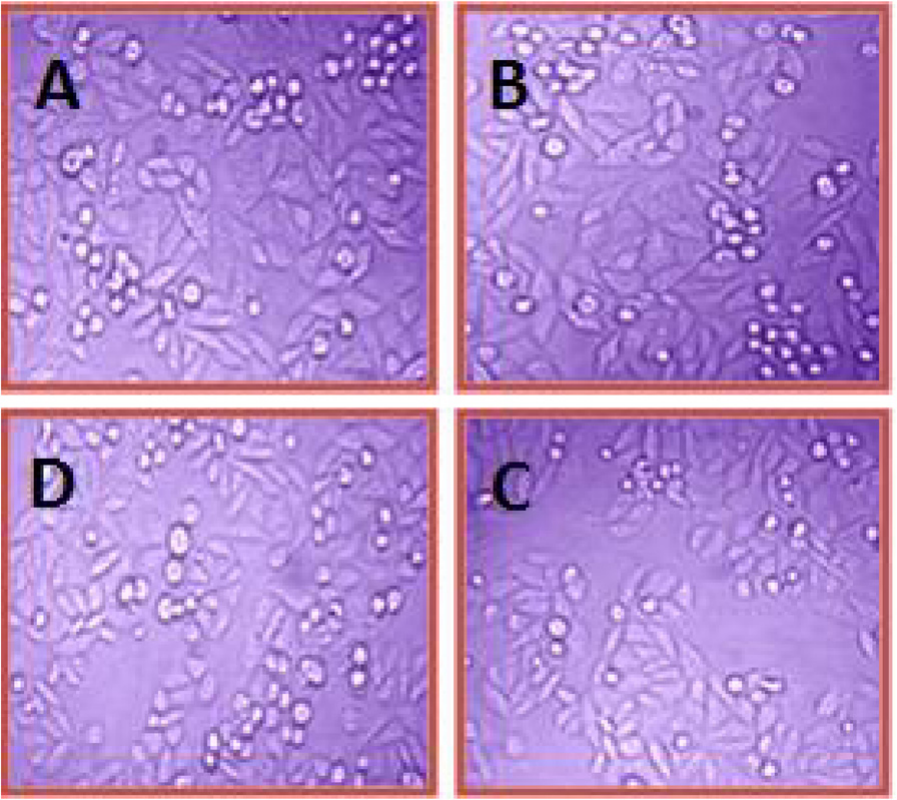
**Continuous culture of SW-48 cells and treatment with SD-208.** The cells were grown as monolayer epithelial-like morphology. SW-48 cells after treatment by DMSO alone as control **(A)**, SD-208 concentrations 0.5 μM **(B)**, 1 μM **(C)** and 2 μM **(D)**.

The *in vitro* growth inhibitory effect of SD-208 was measured by the MTT assay (Roche Applied Science; Germany). This assay is dependent on the ability of viable cells to reduce a yellow tetrazolium salt (MTT) metabolically to a purple formazan product. This reaction takes place when the cell is viable and mitochondrial reductase enzymes are active. Briefly, monolayer cultures were trypsinized in exponential growth phase, and viable cell counts were assessed using trypan blue exclusion. Then, cells were seeded in 96-well flat-bottom microtitration plates (SPL Life Sciences; South Korea) at a density of 5 × 10^4^ cells/well (200 μL media/well). After 24 h, once the cells reached ~85% confluence they were treated with different concentrations of SD-208 (0.5 μM, 1 μM and 2 μM). Following 24 h drug exposure, for the recovery period, the cells were washed two times with fresh and free-FBS medium and the culture continued (Figure 1). Subsequently, fresh medium containing FBS was replaced for removal of efflux and unbound drug. In all *in vitro* experiments, control cells were incubated with dimethylsulfoxide (DMSO) alone (with the final concentration 0.2%). Complete medium was replaced with 100 μl MTT after 48 h incubations. The cells were incubated for 3 h at 37°C then MTT was removed, and 300 μl DMSO were added to each well. Finally, the optical densitometry was measured at a wavelength of 490 nm with background subtraction at 630 nm using a spectrophotometric microplate reader (BioTek Elx 808). The growth inhibition rate was calculated using the following formula:

$$\begin{array}{ll} \;\text{Growth}\;\text{inhibition}\;\text{rate}\;\left(\%\right)=1 & -\left({\text{OD}}_{\text{drug}\;\text{exposure}}/{\text{OD}}_{\text{control}}\right)\;\\ & \times \;100 \end{array}$$

### BrdU assay

The BrdU assay was performed using BrdU ELISA kit according to the manufacturer’s instructions (Roche Applied Science; Germany) for the quantification of cell proliferation base on the measurement of BrdU incorporation during DNA synthesis. Briefly, the SW-48 cells were seeded in 96-well flat-bottom microtitration plates at a density of 5 × 10^4^ cells/well (100 μl media/well). After 24 h at 37°C, the cells were treated with different concentrations of SD-208 (0.5 μM, 1 μM and 2 μM). Every well, except for the background controls, received 10 μl marker solution (1:100 dilution with a sterile medium) and the cells were further incubated for 5 h at 37°C under 5% CO_2_. Removal of the medium from the wells was followed by incubation of the cells in 200 μl FixDenat for 30 min. The FixDenat was removed, and the cells were further incubated for 75 min with the antibody solution. The cells were then washed three times with 200 μl washing buffer (1:10 dilution) and then incubated in 100 μl substrate solution (tetramethylbenzidine) for 15 min. Absorbance was measured at a wavelength of 490 nm using a spectrophotometric microplate reader (BioTek Elx 808). Cells from the same population and treatment that were not BrdU-labeled were used as the background controls. The cell proliferation was calculated using the following formula:

$$\begin{array}{ll} \;\text{Cell}\;\text{proliferation}\;\left(\%\right)= & \left({\text{OD}}_{\text{drug}\;\text{exposure}}/{\text{OD}}_{\text{control}}\right)\\ & \times 100 \end{array}$$

### Animal model implanted with adenocarcinoma cell line (SW-48) and treatment protocol

6–week–old female athymic C56BL/6 nude mice (n = 8 per group) were obtained from Omid Institute for Advanced Biomodels (Tehran, Iran). Animals were kept under optimized hygienic conditions in an individually ventilated cage system. The mice were fed with autoclaved commercial diet and water ad libitum. All animal experiments were carried out according to the Tehran University of Medical Sciences, Ethical Committee Acts and were approved by the TUMS Ethical Committee. In order to establish the xenograft model of SW-48 cell line, the cells were cultured in RPMI 1640 containing 10 percent FBS in 75 cm^2^ cell culture flasks. The cells were trypsinized and harvested. After washing, totally 5 × 10^6^ cells were resuspended and inoculated subcutaneously at a 200 μl volume of serum–free medium into the flank of the animals. Tumor growth was measured twice a week. The volume of tumors was calculated by standard formula (Length × width^2^ × 0.52) and growth curve was drawn (Tomayko, 1989). Xenograft tumors were allowed to reach a size of 100 mm^3^. Then, the animals were randomly divided into two groups of 8 to receive either SD-208 (50 mg/kg/d) or vehicle orally for three weeks. Control animals were received daily drug-free and DMSO-containing deionized water (vehicle). At the end of the treatment period, mice were killed by CO_2_ inhalation and obtained tumors after isolating from animal were fixed in 10% buffered formalin and were subjected to histopathological staining.

### Histopathological diagnosis and immunohistochemistry

Hematoxylin and eosin (H&E) staining for SW-48 tumor confirmation and immunohistochemistry (IHC) staining for Ki-67 and CD34 markers were done. Then, five sections at routine thickness (5 μm) were prepared from the formalin-fixed paraffin-embedded tissue blocks and floated onto charged glass slides (Super-Frost Plus, Fisher Scientific). The slides then were dried overnight at 60°C. For the revision of the histopathological diagnosis and confirmation of developed the SW-48 cell-derived tumors, two hemotoxylin and eosin stained sections were obtained from tissue blocks. Immunohistochemistry carried out for the evaluation of SD-208 effects on proliferation and angiogenesis (Ki-67 and CD34 marker, respectively) in the tumor xenografts. For this, three sections were deparaffinized and hydrated using graded concentrations of ethanol to deionized water from each block. These sections were stained immunohistochemically using three steps-indirect streptavidin method for Monoclonal Mouse Anti-Human Ki-67 Antigen (MIB-1), clone M 7240 (Dako; Denmark) and Monoclonal Mouse Anti-Human CD34, clone QBEnd-10 (Dako; Denmark). Negative controls were obtained by omitting the primary antibody for aforementioned markers under identical test condition. Sections from a lymph node with follicular lymphoid hyperplasia known to be immunoreactive for Ki-67 and CD34 were used as a positive control (as recommended by the manufacturer).

### Statistical analysis

Statistical analysis was performed, and statistical significance of differences between data was evaluated by independent sample Student’s *t*-test for tumor markers (Ki-67 and CD34) and one-way analysis of variance (ANOVA) followed by Tukey’s post tests for multiple comparisons of differences between treatment groups. Data were expressed as mean ± SEM (the standard error of the mean). P values less than 0.05 were considered to indicate statistically significant differences between data sets.

## Results

### *In vitro* effects of SD-208 on SW-48 cell line

**Figure 2:**
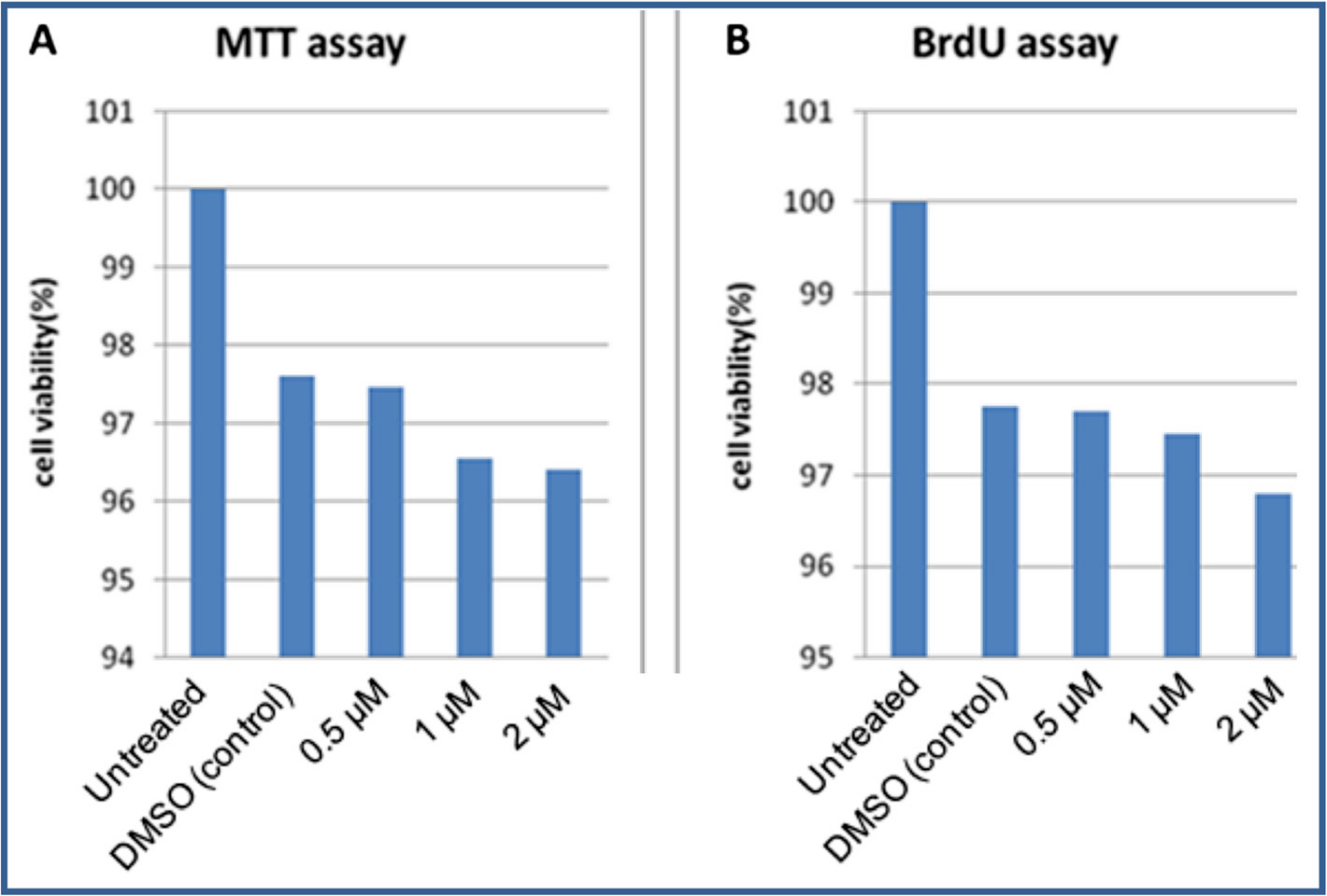
**Effect of SD-208 on the cell growth and proliferation of the SW-48 cells.** SW-48 cells were treated by 0.5, 1 and 2 μM for 48 h. Cell proliferation was examined by MTT and BrdU assays as described in methods. **A**: MTT assay of SW-48 cells after treatment with SD-208 in comparison with controls (untreated and treated with DMSO). **B**: BrdU assay of SW-48 cells after treatment with SD-208 comparison with controls (untreated and treated with DMSO). All data are reported as the percentage change in comparison with the controls, which were arbitrarily assigned 100% cell proliferation. Analysis of one-way ANOVA was used to compare the cell proliferation of SW-48 cells in different concentrations of SD-208 to control. P value < 0.05 was regarded as statistically significant. Results are expressed as the mean ± SEM from three independent experiments.

In order to examine the growth inhibitory effect of SD-208 on CRC, the SW-48 cell line was cultured with different SD-208 concentrations (0.5 μM, 1 μM and 2 μM) for 48 h (Figure 1). As shown in Table 1, evaluation of *in vitro* growth inhibition by MTT (Figure 2A) and BrdU assays (Figure 2B) revealed no significant changes in treated versus untreated cells (P > 0.05).

**Table 1:** Effects of SD-208 on growth of SW-48 cell line

**MTT assay**			
**Inhibition rate (IR)%**	**P value**	**OD value (mean ± SEM)**	**Concentration of SD-208 (μM)**
		2.06 ± 0.085	Untreated
2.36	0.69	1.64 ± 0.098	Control (DMSO)
2.57	0.63	1.61 ± 0.093	0.5
3.4	0.6	1.54 ± 0.099	1
3.64	0.57	1.51 ± 0.098	2
**BrdU assay**			
		2.14 ± 0.079	Untreated
2.24	0.68	1.73 ± 0.081	Control (DMSO)
2.32	0.64	1.71 ± 0.087	0.5
2.6	0.63	1.69 ± 0.097	1
3.2	0.59	1.62 ± 0.098	2

### Colon adenocarcinoma model

**Figure 3:**
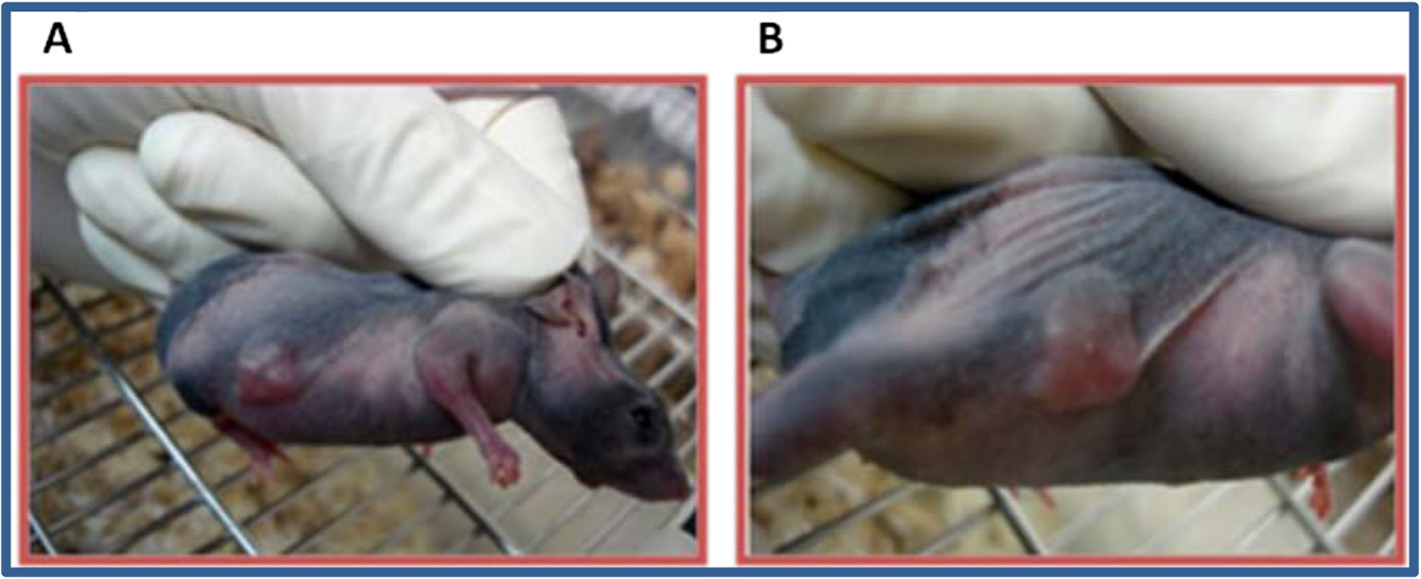
**A representative of colorectal adenocarcinoma model.** Tumor implantation; athymic nude mice implanted by SW-48 cell line, the cells were grown as tumor xenografts after 10 days. Cancer-bearing nude mice were treated with 50 mg/kg/day of SD-208 **(A)** or without SD-208 **(B)**.

**Figure 4:**
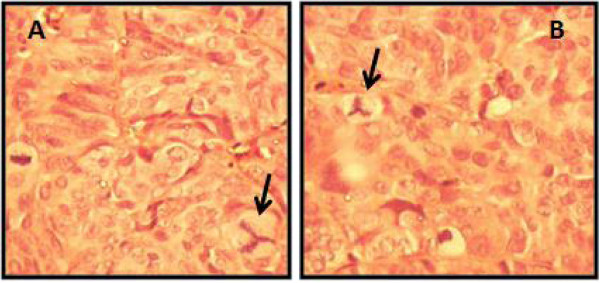
**A representative results of pathological examinations of nude mice tumors with or without SD-208 treatment. A**: tumors of nude mice treated with 50 mg/kg/day stained with H&E. **B**: H&E staining of tumor tissues of control. No significant difference histologically was observed (P > 0.05).

To determine the potential toxicity effects of SD-208, the appropriate numbers of SW-48 cells were injected into nude mice. Subsequently, animals were treated with or without SD-208 (as described in the Methods section). Following SD-208 treatment, we could not observe any changes in animal behavior, body weight and lifespan. These data suggest that SD-208 lacks toxic effects on mice bearing SW-48 tumor (data not shown).Ten days after the subcutaneous inoculation, all 24 nude mice with an observed tumor growth survived with a balanced diet (Figure 3). Based on the standards of cancer-bearing models, 24 mice were selected for further experiments. The mice were sacrificed, and their tumor tissues were excised for pathological examinations.The H&E staining demonstrates marked cellularity with profound hyperchromatism and pleomorphism (Figure 4). Pleomorphic malignant epithelial cells with the clearly nucleolus were visible. In tumor cells, the ratio of nucleus to the cytoplasm was 1/1. Also, high mitotic index and atypical areas were observed in both samples (treated versus controls). The pattern of adenocarcinoma was identical with the human origin in which the cancerous cells were poorly differentiated. Figure 4 indicates H&E staining of tumor tissues from studied animals. These results revealed no major differences in the sizes and the histology of the tumors (P > 0.05).

### Immunohistochemistry

**Figure 5:**
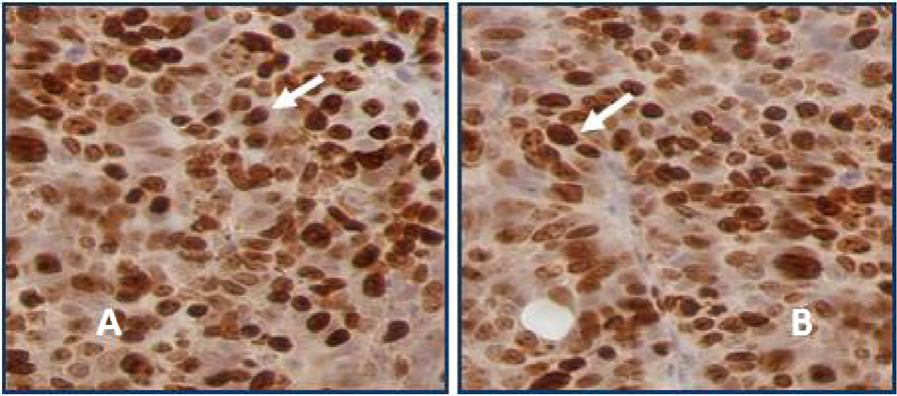
**Colon adenocarcinoma (grade IV) stained immunohistochemically with anti-Ki-67.** Positive immunoreactive showing brown nuclear expression of Ki-67 with a large number (~80%) of stained nuclei reflecting active cellular proliferation in the treated nude mice with SD-208 **(A)** and control **(B)**: Immunohischemical assay showed no significant difference between tests and controls in terms of cellular proliferation (P > 0.05).

**Figure 6:**
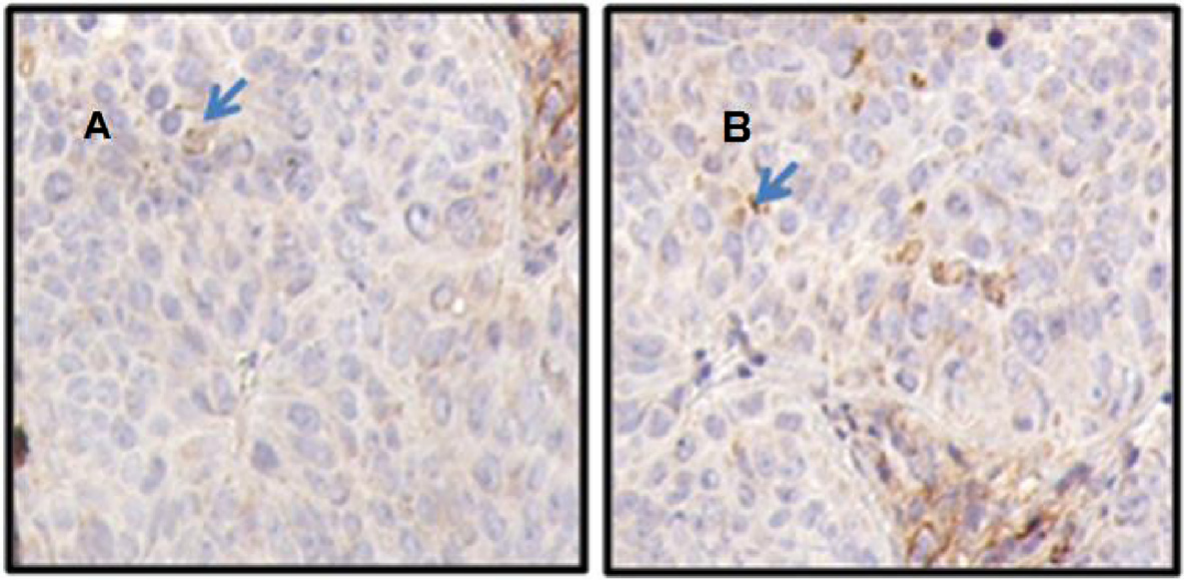
**Colon adenocarcinoma (grade IV) stained immunohistochemically with anti-CD34.** The paraffin embedded sections of tumor tissues were stained with anti-CD34 antibody and positive immunoreactive indicating brown cytoplasmic membrane of endothelial cells and marked microvessel's proliferation. Microvessel density (MVD) was ~40 microvessel/mm^2^. SD-208-treated tumors **(A)** vs controls **(B)** revealed no significant immunohistologically differences (P > 0.05). Note the prominent vascularity. Arrows indicate CD34 staining of the cytoplasmic membrane.

Immunohistochemical analysis was conducted using Ki-67 and CD34 antibodies. Immunoreactive SW-48 cells to anti- Ki-67 are brown nuclear with a diffuse pattern. It was scored by counting about 1000 cells in 10 different fields. Each brown stained nucleus was considered positive, regardless of intensity (Figure 5). Immunohistochemical staining of treated and untreated nude mice’s tissues using anti-CD34 (as shown in the Figure 6) showed no significant changes among studied samples.

### Ki-67 and CD34 expressions in the tumor xenografts

The results from the immunochemical assay indicated that Ki-67 was predominantly expressed in the nucleus. As already pointed out, the immunohistochemically stained tumors with anti-Ki-67 showed positively brown nuclear in the studied tissues (Figure 5). The Ki-67 expression levels between the test and control groups did not show significant changes (P > 0.05). Immunohistochemical staining using anti-CD34 antibody on tumor tissues from either treated or untreated nude mice with SD-208 showed cytoplasmic membrane of endothelial cells in brown color and marked microvessels proliferation (Figure 6).

## Discussion

Despite aggressive surgery, radiotherapy and chemotherapy, treatment of malignant CRC remains formidable. Even though that TGF-β suppresses proliferation of certain carcinoma cells and is well-known to be a tumor suppressor, it promotes tumor development, progression and metastasis in human cancers including CRC, glioma, osteosarcoma, breast, lung and pancreatic cancers [14-16]. The insights into the role of the TGF-β signaling in carcinogenesis importantly have come due to experimental study on animal models. Understanding the mechanisms by which TGF-β signaling regulates tumor development and progression is critical for designing the beneficial therapeutic strategies for the cancers [14,16-18]. In the last few years, because of availability and easily drug delivery, the targeting of receptor kinases by small-molecule inhibitors has been a profound area of experimental cancer studies [16,17,19].

It is thought that pharmacological blockade of the TGF-β signaling pathway by TGβRI inhibitors may be as a potential strategy for cancer therapy [17-19]. For the first time, we evaluated the efficacy of systemic targeting of the TGF-β pathway by a TGβRI kinase inhibitor, SD-208, on a high-grade colon adenocarcinoma cell line, SW-48, *in vitro* and in developed heterotopic colon tumors in model that shares similarities with human colon cancer. Our findings using MTT and BrdU assays demonstrated that treatment of SW-48 cells with SD-208 using different doses had no significant inhibitory effects on cell growth and proliferation. Our *in vitro* results were consistent with one study revealing SD-208 can not reduce viability or proliferation of human malignant glioma cells [17]. However, this study showed that SD-208 regulates the growth of glioma in syngeneic mice without changes in proliferation, apoptosis or angiogenesis. Also, other investigators demonstrated that SD-208 failed to inhibit R3T tumor growth or metastasis in athymic nude mice [20]. On the other hand, some studies suggested that the various kinase inhibitors including SD-208, were able to inhibit TGF-β-evoked migration and invasion [20-22]. These results indicated that reduction of tumors was due to regulation of immune surveillance and importantly correlated with tumor-reactive immune regulation and increased immune infiltration [21,22]. Thereby, it has been commented that SD-208 could be a promising agent for the treatment of human malignancy and other conditions associated with pathological TGF-β activity.

In addition, we revealed that when drinking water contains SD-208 daily, the drug could not inhibit the growth of established heterotopic SW-48 tumors and its progression in nude mice. Further histological analysis indicated that SD-208 had no significant effects on proliferation (Ki-67 positivity) or the number of blood vessels and angiogenesis (CD34 positivity). This is consistent with reports by others showing SD-208 had no effect on the growth of primary and metastatic R3T mammary tumors in athymic nude mice [18]. In the agreement with our results and previous studies, it has been shown that an inhibitor of TGF-β receptor 1 kinase, SM16, lost efficacy against the AB12 mesothelioma model in SCID mice [21].

Also, we found that SD-208 is not effective in limiting heterotopic tumor growth and not proper to treat established primary tumors settings at least in SW-48 colorectal cancer cells. The failure of SD-208 to inhibit SW-48 tumor growth in nude mice suggests that the suppression of angiogenesis and proliferation could be dependent on cell lineage and cell context [23]. Our results are not the first report suggesting the inability of SD-208 to inhibit angiogenesis and proliferation. Some researchers reported that SD-208 was unable to cause differences in microvessel density in gliomas [17]. Furthermore, a study of two TGF-βR1 inhibitors, SD-093 and SD-208, on two murine mammary carcinoma cell lines (R3T and 4 T1) revealed that SD-208 failed to inhibit R3T tumor growth or metastasis in athymic nude mice. However, SD-208 treatment led to a reduction in microvessel density in mammary tumors [20].

It is quite understandable that the efficacy of this kinase inhibitor is a controversial issue in cancer treatment [20]. In this regard, several scenarios could be proposed to elucidate the failure of SD-208 as an anti-tumor: (1) genetic alterations such as chromosomal abnormalities and (2) gene mutation in any TGF-β signaling pathway.

## Conclusion

We have demonstrated that SD-208, a TGβRI kinase inhibitor, was not able to reduce tumor growth and angiogenesis in a heterotopic human colorectal cancer model. Our results also for the first time indicated that this anti-cancer agent could not potentially have a therapeutic benefit in at least proportion of CRC. To get more insight into the potential biological effects of SD-208 on CRC, different CRC cells with various differentiated status are required to treat with SD-208. Moreover, because of the complexity of TGF-β signaling role and crosstalk with other factors (such as immunochemotactic and angiogenic agents) in the derived tumor microenvironment, designing therapeutic intervention strategies for targeting tumors in this field, should be very carefully.

## Abbreviations

TGF-β: Transforming growth factor-β; CRC: Colorectal cancer; TGβRI or TβRI: TGF-β receptor 1, TGβRII or TβRII, TGF-β receptor 2; SW-48: A colon adenocarcinoma cell line; SD-208: A TGF-β receptor I kinase inhibitor; MTT: Methyl thiazolyl tetrazolium; BrdU: Bromo-2′-deoxyuridine; H&E: Hematoxylin and Eosin; IHC: Immunohistochemistry.

## Competing interests

The authors declare they have no competing interests.

## Authors’ contributions

MH and AA contributed to idea and study design; ARD contributed to the supervision of sections of the study. MHG, SHG, GRM and FS assisted with cell culture study and experimentation and provided scientific advice, MA assisted with analysis of the data. SM and SA assisted with IHC staining and data analysis. AA prepared the manuscript which SM, MH and AK significantly revised. All authors read and approved the final manuscript.
